# Where teachers are few: documenting available faculty in five Tanzanian medical schools

**DOI:** 10.3402/gha.v9.32717

**Published:** 2016-10-13

**Authors:** Charles A. Mkony, Ephata E. Kaaya, Alex J. Goodell, Sarah B. Macfarlane

**Affiliations:** 1 Department of Surgery, Muhimbili University of Health and Allied Sciences, Dar es Salaam, Tanzania; 2 Department of Pathology, Muhimbili University of Health and Allied Sciences, Dar es Salaam, Tanzania; 3 School of Medicine, University of California San Francisco, San Francisco, CA, USA; 4 Global Health Sciences, University of California San Francisco, San Francisco, CA, USA

**Keywords:** teaching faculty, academic staff, universities, Tanzania, teaching, health, workforce, medical education

## Abstract

**Background:**

Faced with one of the lowest physician-to-population ratios in the world, the Government of Tanzania is urging its medical schools to train more physicians. The annual number of medical students admitted across the country rose from 55 in the 1990s to 1,680 approved places for the 2015/16 academic year. These escalating numbers strain existing faculty.

**Objective:**

To describe the availability of faculty in medical schools in Tanzania.

**Design:**

We identified faculty lists published on the Internet by five Tanzanian medical schools for the 2011/12 academic year and analyzed the appointment status, rank, discipline, and qualifications of faculty members.

**Results:**

The five schools reported 366 appointed faculty members (excluding visiting, part-time, or honorary appointments) for an estimated total enrolled student capacity of 3,275. Thirty-eight percent of these faculty were senior lecturers or higher. Twenty-seven percent of the appointments were in basic science, 51% in clinical science, and 21% in public health departments. The most populated disciplines (more than 20 faculty members across the five institutions) were biochemistry and molecular biology, medicine, obstetrics and gynecology, pediatrics, and surgery; the least populated disciplines (less than 10 faculty members) were anesthesiology, behavioral sciences, dermatology, dental surgery, emergency medicine, hematology, ophthalmology, orthopedics, otorhinolaryngology, oncology and radiology, psychiatry. These figures are only indicative of faculty numbers because of differences in the way the schools published their faculty lists.

**Conclusions:**

Universities are not recruiting faculty at the same rate that they are admitting students, and there is an imbalance in the distribution of faculty across disciplines. Although there are differences among the universities, all are struggling to recruit and retain staff. If Tanzanian universities, the government, donors, and international partners commit resources to develop, recruit, and retain new faculty, Tanzania could build faculty numbers to permit a quality educational experience for its doctors of tomorrow.

## Introduction

Tanzania has one of the lowest physician-to-population ratios in the world (1) despite high poverty-related disease burdens. To address this shortage, the Government of Tanzania has called on its medical schools to train more physicians, and universities are rapidly expanding their enrolments (2). The number of admissions to medical schools increased from 55 student admissions in 1991 to 1,680 admissions approved by the Tanzania Commission for Universities (TCU) for 2015/16 (3). There has not been a parallel drive to recruit and train faculty qualified to educate the increasing numbers of medical students. A 2009 audit of medical schools by the East African Community Partner States National Medical and Dental Practitioners Regulatory Boards/Councils reported shortfalls of faculty in the region; in Tanzania, the review highlighted particular shortages in the basic sciences and recommended that some universities significantly reduce their student intakes until they had recruited more teachers (4). New schools continue to open, further escalating the number of enrolled students.

Tanzania, like other African countries, faces a dilemma. If universities admit larger cohorts without training and employing more teachers, the standards of medical education will deteriorate and students may not learn how to provide quality care. Yet by halting expansion of student admissions, schools limit availability of professional care for large segments of the growing population and restrict the pool of potential faculty recruits.

Tanzanian students enter medical school straight from high school and need faculty to serve as role models and mentors whom they can come to know personally. In a few years, these students will run district hospitals, operate on birthing mothers in crisis whose lives depend on them, and confront disease outbreaks, perhaps where immunization coverage is too low, all with little supervision. Yet at Muhimbili University of Health and Allied Sciences (MUHAS), for example, with the largest medical faculty in Tanzania, students’ needs for preparation are often unmet. They may face overcrowded classrooms, share dissection of a cadaver with a dozen or more fellow students, compete for a space around a patient's bedside, or observe surgical procedures from a distance. Leon et al. noted in a 2005 survey of 130 Tanzanian medical students that two thirds reported being less motivated to pursue medicine at the end of their studies compared to when they began (5). Trainees need more face-to-face faculty attention if they are to take up leadership positions within health systems or become faculty in academic centers. Students are also ill-prepared to serve those in rural areas, where the majority of the population resides. Recent generations of medical students – many of whom have an urban background and little experience living in remote villages with minimal communication, transportation, or other urban amenities – are confronted with difficulties when they accept postings in rural areas. The educational challenges for medical students in Tanzania are many and broad.

In 2012, we attempted to understand the national situation by reviewing publicly available information about teaching capacities in Tanzanian medical schools. We examined the total numbers and distribution of faculty across the basic, clinical, and public health sciences by rank and qualification. At that time, the TCU had accredited seven institutions to admit 825 medical students for the 2011/12 academic year (Table 1) (6). Four years later, five additional universities had opened, and the TCU had approved them to admit 1,680 medical students for the 2015/16 academic year (Table 1) (3). Given these dramatic increases in student numbers, we revisited the 2012 figures to assess the strains on limited faculty that this rapid increase in numbers might pose. Although the quality of publically available information is limited, our findings provide some insight into the medical teaching capacity across the majority of schools in an entire African country – a perspective that, to the best of our knowledge, has not been previously described.

**Table 1:** Medical schools in Tanzania and their admission capacities approved by the Tanzania Commission for Universities for the academic years 2011/12 (6) and 2015/16 (3)

					Medical student admission capacity^a^	
					
Name	Acronym	Type	Location	Year of first admission	2011/12	2015/16
Muhimbili University of Health and Allied Sciences^b^	MUHAS	Public	Dar es Salaam	1963	200	225
International Medical and Technological University^b^	IMTU	Private	Dar es Salaam	1997	180	180
Kilimanjaro Christian Medical College, Tumaini University^b^	KCMC	Private	Moshi	1997	100	170
Hubert Kairuki Memorial University^b^	HKMU	Private	Dar es Salaam	1998	50	175
Catholic University of Health and Allied Sciences^b^	CUHAS	Private	Mwanza	2003	125	185
University of Dodoma College of Health Sciences	UDOM	Public	Dodoma	2009	120	175
St. Francis University College of Health and Allied Sciences, Saint Augustine University of Tanzania	SFUCHAS	Private	Ifakara	2010	50	120
Kampala International University	KIU	Private	Dar es Salaam	2012	–	100
State University of Zanzibar	SUZA	Public	Zanzibar	2013	–	50
Archbishop James University College	AJUCO	Private	Songea	2015	–	100
University of Dar es Salaam	UDSM	Public	Dar es Salaam	2015	–	100
St. Joseph College of Health Sciences	SJCHS	Private	Dar es Salaam	2015	–	100
Total annual admission capacity					825	1,680

a The number of medical students that the Tanzania Commission for Universities accredited each institution to admit for the corresponding academic year.

b An institution in this study.

## Methods

We searched the Internet for documents that contained faculty lists for the seven schools in Tanzania that were admitting students in the academic year 2011/12. We found faculty lists from 2011 to 2012 prospectuses for the medical schools at MUHAS, CUHAS, and KCMC; and on websites accessed in June 2012 for medical schools at HKMU and IMTU. We could not find faculty lists for UDOM and SFCUHAS – both were newer medical schools that had not yet graduated any students and that were still recruiting faculty. We transcribed into Excel the names of faculty members along with their degrees, ranks, and departments for each of the five institutions. Because other medical schools had integral departments of public health, we included faculty listed in MUHAS's prospectus for its separate School of Public Health and Social Sciences.

### Classification of faculty status

Universities in Tanzania confer faculty status through appointments made by their university senates. These appointments can be supplemented by: *honorary lecturers* who have senate appointments but are not paid; *part-time lecturers* who usually are invited to give lectures on specific topics and are paid by the hour; and *visiting faculty* who may be scientists working for national research institutes, faculty from other medical schools or universities with science programs, retired faculty, experts working for non-governmental organizations, non-academic clinicians working at associated teaching hospitals, or who are faculty visiting Tanzania from collaborating international institutions. We designated faculty members as *appointed* if their names were not qualified with the term part-time, visiting, or honorary. Some MUHAS faculty teach on a part-time basis at other schools. When the name of MUHAS-appointed faculty appeared on a list for another school, we excluded that name from the total faculty for that school, even if the faculty member was not identified as part-time, visiting, or honorary.

### Classification of faculty by rank

In Tanzania, faculty ranks range from tutorial assistant, assistant lecturer, lecturer, senior lecturer, associate professor, to full professor (Table 2).The minimum requirement to be appointed as a lecturer is a PhD in the basic sciences or in public health or a Master of Medicine (MMed) in an appropriate clinical specialty. In basic science and public health, the universities may appoint some promising master's degree graduates as assistant lecturers, and some bachelor's degree graduates as tutorial assistants, with the expectation that each will seek funding and obtain a PhD to become ‘career faculty’. We classified faculty as: 1) *junior* (tutorial assistants and assistant lecturers); 2) *mid-level* (lecturers); and 3) *senior* (senior lecturers, associate professors, and full professors); and we describe all mid-level and senior faculty as *career faculty* (Table 2). We classified individuals as having a PhD or equivalent if the prospectus indicated they had a PhD, DrPH, DSc, ScD, or DPhil.

**Table 2:** Ranks of faculty in medical schools in Tanzania

Faculty rank	Required degree	Classification used in this study	
Tutorial assistant	Bachelor's	Junior faculty	
Assistant lecturer	Master's		
Lecturer	MD and MMed for clinical faculty; or PhD for non-clinical faculty	Mid-level faculty	Career faculty
Senior lecturer Associate professor Full professor		Senior faculty	

### Classification of faculty by departments

We classified the departments as follows: 1) *basic science*: pathology, pharmacy, and physiology; 2) *clinical science*: anesthesiology, dental surgery, dermatology, emergency medicine, hematology, medicine, obstetrics and gynecology, oncology and radiology, ophthalmology, orthopedics and traumatology, otorhinolaryngology, pediatrics and child health, psychiatry, and surgery; and 3) *public health*: behavioral sciences, community health, development studies, environmental and occupational health, epidemiology and biostatistics, parasitology and entomology (traditionally within public health in Tanzania).

### Calculation of student numbers

We could not find the actual numbers of students admitted by each institution in 2011/12. Instead we used the ‘admission capacity’ or the numbers of students the Tanzanian university accrediting body, the TCU, had approved each institution to admit. These figures, totaling 655 students, would have approximated the number admitted at that time (Table 1). To calculate the total numbers of enrolled students, we multiplied the admission capacity by five (the number of years of medical training in Tanzania), resulting in a total of 3,275 enrolled students across the five schools. This would have been an overestimation of the actual number enrolled because institutions increased their enrollment numbers over the years up to 2011/12. However, these estimated total enrollment numbers represent the expected numbers if admissions continued at the same rate as the 2011/12 admission capacity.

## Results

### Numbers and status of faculty

**Fig. 1:**
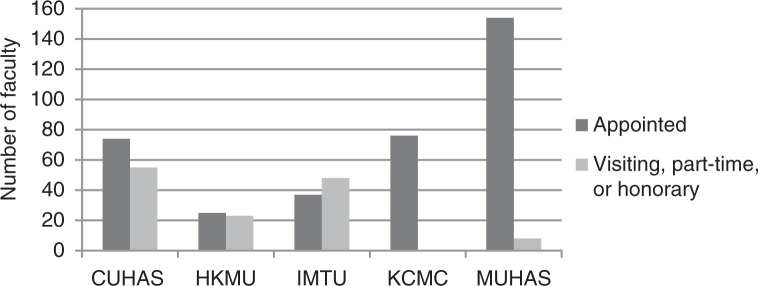
Distribution of 366 appointed faculty and 134 visiting, part-time, and honorary faculty for five medical schools in Tanzania, 2011/12.

The five institutions listed 500 faculty among them. Of these, CUHAS listed 55 (43%) out of 129 faculty as visiting; HKMU listed 23 (48%) out of 48 as part-time; IMTU listed 48 (56%) out of 85 as visiting; KCMC listed 76 faculty without specifying any as visiting, part-time, or honorary; and MUHAS listed eight (5%) out of 162 as honorary lecturers (Fig. 1). Although visiting, part-time, and honorary faculty represent a valuable pool of teachers from which universities can draw, because the universities did not report them uniformly and we did not know their teaching contributions, we restricted our remaining analyses to the 366 faculty appointed by the five schools. Of the 366, MUHAS, with 154 faculty (including seven on study leave or leave of absence), accounted for 42% of all positions, and the other schools had between 25 and 76 faculty members each.

### Distribution of faculty by department type

**Fig. 2:**
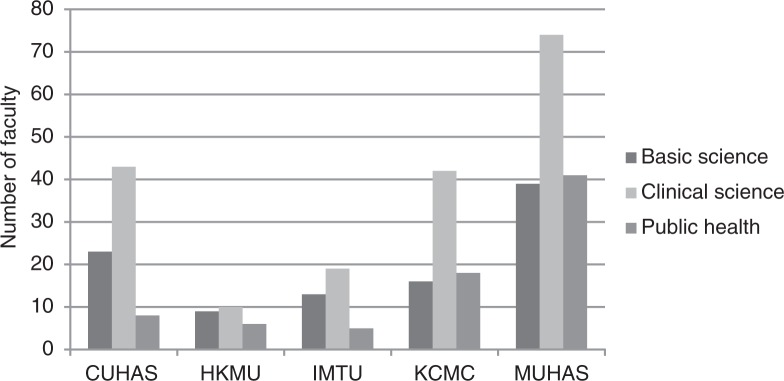
Distribution of 366 appointed faculty by speciality for five medical schools in Tanzania, 2011/12.

Figure 2 shows the distribution of faculty by department type and institution. Twenty-seven percent of the 366 appointments were in basic science, 51% in clinical science, and 21% in public health departments. The total number of appointed faculty in basic science was 100 (64 of whom were career faculty), ranging from nine to 39 per institution; in clinical science was 188 (165 of whom were career faculty), ranging from 10 to 74 per institution; and in public health was 78 (43 of whom were career faculty), ranging from five to 41 per institution.

**Fig. 3:**
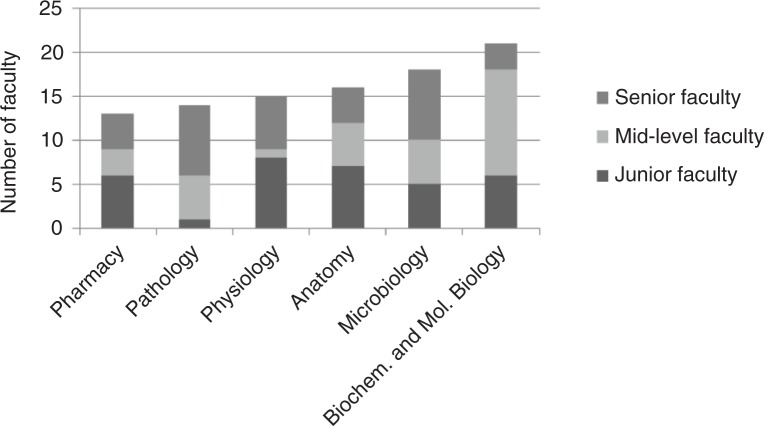
Distribution of 97^a^ appointed basic science faculty by academic rank for five medical schools in Tanzania, 2011/12. ^a^The academic rank of three faculty members was missing. MUHAS has a school of pharmacy whose numbers were not included in the study.

**Fig. 4:**
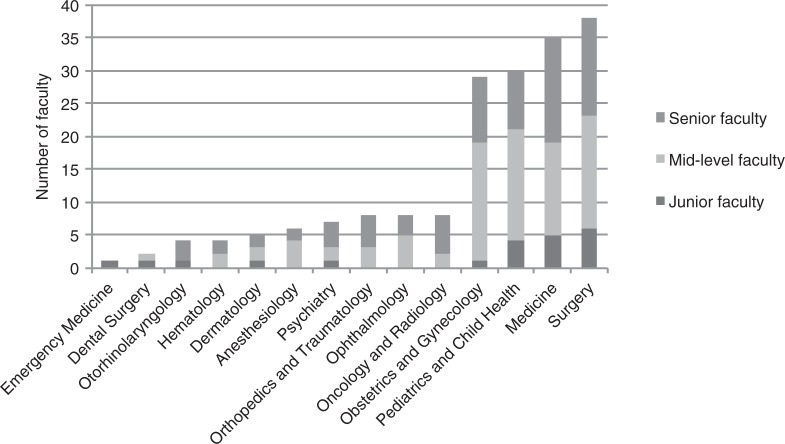
Distribution of 185^a^ appointed clinical science faculty by academic rank for five medical schools in Tanzania, 2011/12. ^a^The academic rank of one faculty member was missing, and two lacked primary departments. MUHAS has a school of dentistry whose numbers were not included in the study.

**Fig. 5:**
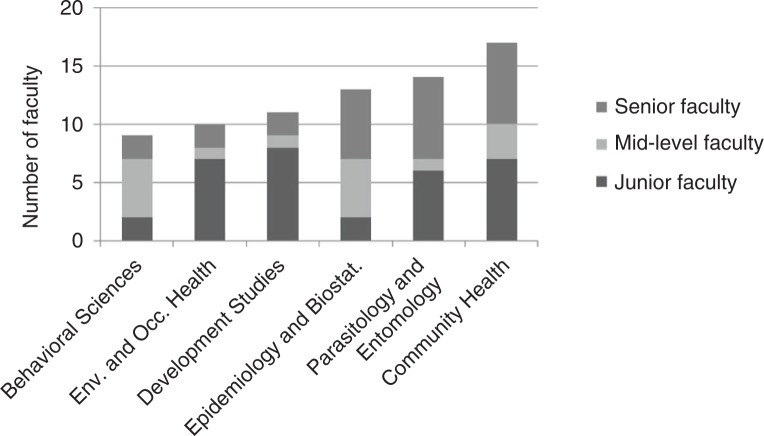
Distribution of 74^a^ appointed public health faculty by academic rank for five medical schools in Tanzania, 2011/12. ^a^The academic rank of three faculty members was missing, and one lacked a primary department.

Figs. (3–5) show the distributions of faculty across major disciplines. The most populated disciplines (more than 20 faculty in total) were biochemistry and molecular biology, medicine, obstetrics and gynecology, pediatrics, and surgery. The least populated disciplines (less than 10 faculty in total) were anesthesiology, behavioral sciences, dental surgery, dermatology, emergency medicine, hematology, oncology and radiology, ophthalmology, orthopedics, otorhinolaryngology, and psychiatry. The average department size varied from two to seven across the institutions, with the smallest consisting of only one person and the largest (medicine at MUHAS) consisting of 15 faculty.

### Distribution of faculty by rank and advanced degree

**Fig. 6:**
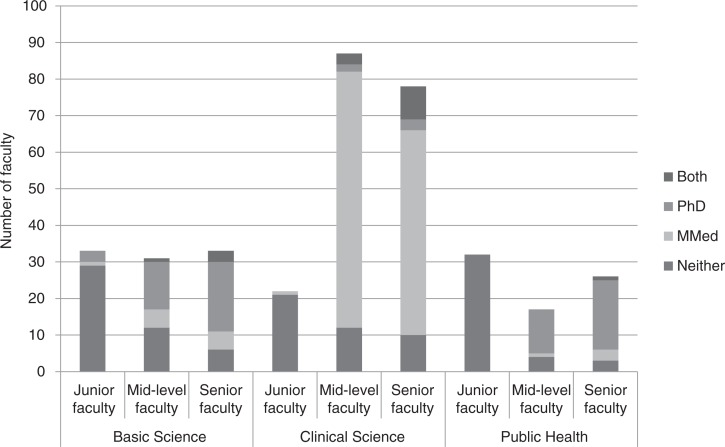
Distribution of advanced degrees among 359^a^ faculty by rank and discipline for five medical schools in Tanzania, 2011/12. ^a^The academic rank of seven faculty members was missing.

Fifty-six percent of MUHAS's appointed faculty was senior compared to 36% at HKMU, 25% at IMTU, 24% at KCMC, and 22% at CUHAS. The proportion of senior faculty was similar across department types, averaging 38%, but with differences in the distributions of junior faculty that reflect different recruitment patterns. We found as many junior as mid-level faculty in the basic sciences; clinical sciences had very few junior faculty, and public health had the highest proportion of junior faculty (Fig. 6). Ten percent of career faculty in the clinical sciences had an advanced degree compared to 56% in the basic sciences and 74% in public health. Eighty-six percent of career faculty had an MMed, an advanced research degree, or both (Fig. 6).

### Ratios of enrolled student numbers to faculty

Table 3 shows the ratios of estimated total number of enrolled students to faculty numbers. The five schools had between 26 and 69 enrolled students per basic science faculty member, averaging 33 students per faculty member; between 12 and 47 enrolled students per clinical science faculty, averaging 17 students per faculty member; and between 24 and 180 enrolled students per public health faculty, averaging 42 students per faculty member. IMTU had the highest number of enrolled students per faculty member in all department types. Overall, across departments, the numbers of enrolled students per faculty member ranged from six (for MUHAS) to 24 (for IMTU), with an average of nine across all five schools (Table 3).

**Table 3:** Estimated enrolled student-to-faculty ratios by department type for five medical schools in Tanzania, 2011/12

School	Basic Science Ratio (no. of faculty)	Clinical Science Ratio (no. of faculty)	Public Health Ratio (no. of faculty)	Overall Ratio (no. of faculty)	Estimated total number of enrolled students^a^
CUHAS	27:1 (23)	15:1 (43)	78:1 (8)	8:1 (74)	625
HKMU	28:1 (9)	25:1 (10)	42:1 (6)	10:1 (25)	250
IMTU	69:1 (13)	47:1 (19)	180:1 (5)	24:1 (37)	900
KCMC	31:1 (16)	12:1 (42)	28:1 (18)	7:1 (76)	500
MUHAS	26:1 (39)	14:1 (74)	24:1 (41)	6:1 (154)	1,000
Overall	33:1 (100)	17:1 (188)	42:1 (78)	9:1 (366)	3,275

a Estimated total enrolled student numbers were calculated as five years of intake at the admission capacity published by the Tanzania Commission for Universities for 2011/12 (6). These figures likely were higher than the actual numbers because student enrolment was lower for the years before 2011/12.

### Limitations of the findings

There were clear limitations to our approach of using publically available information. These limitations included:

1. The published lists may not have represented exactly the same time periods for each university.
2. There were differences in terminology: schools other than MUHAS listed some faculty in their medical schools as members of departments of public health, pharmacy, or dentistry. MUHAS has separate schools for these professions; we only included faculty from MUHAS's School of Public Health in this study. Some schools may have listed specialty faculty (for example, in anesthesiology or dermatology) simply under ‘medicine’, thereby under-representing the number of specialists.
3. There were inconsistencies in the way universities categorized faculty: some faculty may not have been listed as visiting or part-time when they were (for example, KCMC did not list any such faculty). MUHAS does not classify as faculty any clinicians at the Muhimbili National Hospital who teach its students informally, whereas the others schools may have listed hospital clinicians as faculty.

## Discussion

Over a four-year period from 2011/12 to 2015/16, the student admission capacity across all schools in Tanzania rose by about 50% from 825 to 1,680. If medical school recruitment continues at this rate, Tanzania will meet the recommended minimum of one physician per 10,000 population before 2025 (7). The challenge is whether these graduates will be adequately trained to provide quality care to their patients and whether they will be sufficiently inspired and supported by their education to stay in clinical practice in Tanzania. A 2012 study tracked 2,246 Tanzanian medical doctors and found that only 1,262 of them were clinically active in Tanzania (8). The remainder were unemployed or suspended after striking (294), working for non-governmental organizations (276), in teaching or research institutions (191), abroad (94), undertaking further studies (63), working in health-related administrative positions (50), or working outside the health sector (16).

The quality of the students’ education depends primarily on the number and capacity of faculty and how their institutions facilitate their teaching. This review only examines numbers and types of faculty in relation to student numbers and does not address teaching quality. The findings, however limited, provide some indication of the pressures placed on faculty and educational institutions by the rapidly expanding enrolment of students.

It is important to set the numbers in context. Beginning in 1971, the national enrolment was 50 students per year in one medical school in the University of Dar es Salaam (which later became MUHAS) (9). The four private schools included in this study (IMTU, KCMC, HKMU, and CUHAS) opened between 1997 and 2003. Between 2009 and 2015, three public schools (UDOM, UDSM, and SUZA) and four private schools (SFUCHAS, AJUCO, KIU, and SJCHS) opened, bringing the annual national admission capacity to 1,680 in 2015/16 (Table 1). MUHAS archives (accessed by CM) indicated that, in 1996, 155 faculty at one institution supervised an entering class of 55 students (with a total enrolled student capacity of 275). This review finds that, 15 years later, around 366 faculty across five institutions supervised an entering class of 655 students (with an estimated total enrolled student capacity of 3,275). This represents a change in the student-to-faculty ratio from 1.8 to 8.9 enrolled students per faculty member. Even allowing for large errors in the review data, the change is striking – especially knowing that the entry numbers for these institutions are still rising. The TCU approved 935 admissions for these five institutions in 2015/16. Some schools relied heavily on visiting and part-time faculty, which we excluded from the analyses. These faculty can be an invaluable additional resource depending on the time they are able to commit to teaching.

In 2015, we attempted to update the findings but were only able to find recent faculty lists for four of the five schools. CUHAS's 2014/15 prospectus indicated that it had increased its faculty from 74 to 100. MUHAS's 2014/15 prospectus indicated that it had increased its faculty from 154 to 185 (including seven and 17, respectively, on study leave or leave of absence). IMTU's webpage provided a faculty list dated 2013/14 that indicated it had increased its faculty from 37 to 59, while HKMU's webpage accessed in May 2015 indicated a change from 25 to 24 faculty. In total, the four schools had added 78 faculty members since 2011/12, while they planned to admit 210 additional students annually starting in 2015/16. If these four schools maintain this level of student recruitment over the next five years with the same number of teaching faculty, their total enrolled student-to-faculty ratio would remain at approximately 10, as in 2011/12.

In our analysis of the 2011/12 data, the number of appointed faculty per institution ranged from 25 to 154 (Table 3), which is in the same range as other African countries. Chen et al., for example, reported an average of 153 (SD=257) teaching staff across 98 responding African medical schools and only two schools with teaching staff of more than 700 (10). On initial assessment, ratios of 10 or less students per faculty member look quite impressive, but these institutional figures are deceptive. The ratios ranged from between 26 and 69 students per faculty member for the basic sciences, between 12 and 47 for the clinical sciences, and between 24 and 180 for public health. Furthermore, most faculty members have major clinical, research, and administrative duties. Each department, however small, requires a head, and each school requires a dean and directors, all selected from the few faculty often unsupported by a strong institutional administrative structure. Additionally, undergraduate teaching of doctors-to-be may not account for the highest teaching load. At MUHAS, for example, the same faculty members are responsible for teaching students in 65 master's programs and also for supervising PhD students. It is by providing postgraduate programs such as these that schools prepare the next generation of faculty and clinicians.

It is important also to look at the composition of the faculty by institution and across the system. Nearly 40% of faculty members was senior. Because promotion is slow in Tanzania, this means that most senior faculty are nearing the end of their careers or have retired and returned to service on short contracts. Working for a public institution, MUHAS faculty are obliged by the government to retire at age 60, at which time they either seek employment at a private university or – if they are senior faculty – MUHAS re-employs them with short-term contracts until they reach 70 years of age. Administrative records for MUHAS in June 2016 (accessed by CM), for example, indicated that 8.6% of MUHAS faculty was retired and hired back on short-term contracts and 13.6% of the remainder was due to retire within five years.

Almost 60% of MUHAS faculty members was senior, compared to less than 40% for the other four institutions. This is mainly because MUHAS began employing faculty 34 years before the other schools opened. The comparison of rank among schools also depends on each institution's combination of disciplines because there are differences in recruitment policies among basic science, clinical and public health departments. Nationwide, junior faculty for the clinical sciences are drawn from MMed students who do some teaching as students but are not appointed by the schools, whereas public health and basic sciences appoint tutorial assistants and assistant lecturers to prepare them to become career faculty. It appears that, in 2011/12, MUHAS had not recently appointed junior faculty at the same rate as the other universities. A 10-year freeze on employment beginning in 1992 (11) and the current requirement for public universities to obtain permits from the Public Service Commission to employ faculty have both interfered with MUHAS's ability to recruit new faculty.

The numbers of faculty per discipline across the five institutions ranged from one to 38, and the average department size ranged from one to seven. The disciplines most populated with faculty were medicine, obstetrics and gynecology, pediatrics, and surgery – all specialties in which Tanzanian schools admit larger numbers of MMed students (who may become future faculty) – than for other MMed programs. This distribution was not unexpected because these were core specialty programs developed very early when Tanzania started to train specialists locally, probably reflecting the health system needs of the country. The socioeconomic environment in Tanzania tends to attract faculty to clinical disciplines because they can earn additional income through private clinical work. Public health disciplines have some attraction for young professionals who may prefer regular working hours. Basic sciences may be relatively less attractive because schools require medical graduates to obtain PhDs before their appointment as career faculty, and because there are few opportunities for income beyond the faculty member's salary.

In 2016, the East African Community Partner States National Medical and Dental Practitioners Regulatory Boards/Councils audit expressed concern once again about faculty shortages in Tanzania and recommended that even MUHAS, with the highest number of faculty, either increase its faculty numbers or lower its student intake to attain a more favorable student-to-faculty ratio. This audit assessed the schools using TCU recommendations that the student-to-faculty ratio must range between 8:1 and 20:1. The upper limit of this range is higher than international figures, for example, in 2016, the top 30 medical schools in the UK had student-to-faculty ratios of between 4.7:1 and 11:1 (12). In 2011/12, only one of the five Tanzanian schools (IMTU) lay outside the TCU recommended range. However, to maintain a balanced education for their medical students, schools need enough faculty across the disciplines. Whether or not their overall ratios fall within the TCU recommended range, schools cannot teach the basic sciences to large numbers of students without sufficient faculty. If each of the basic sciences, clinical sciences, and public health were to at least reach a ratio of 20:1, then in 2011/12 basic sciences would have required 64 additional faculty, clinical sciences would have met the target, and public health would have required 86 additional faculty across the five schools. For 2015/16, Table 1 indicates a potential intake of 1,680 students, that is, 8,400 enrolled students (over the next 5 years). Applying the same ratio of at least 20:1 would require 420 faculty in each of the basic sciences, clinical sciences, and public health, making a total of 1,260 faculty across 12 schools and representing a large pool of new faculty.

## Conclusions

Despite the limitations of using publicly available data, this study provides the most comprehensive picture yet of the medical faculty situation in an African country. As rapid population growth accelerates the need for doctors and the demand for university places in Tanzania and elsewhere in Africa, student-to-faculty ratios are deteriorating. Between 2011/12, when we undertook this study, and 2015/16, seven schools have opened in Tanzania and the approved student admission capacity has doubled. The need for faculty to teach these students has also escalated. The low numbers of faculty and particularly the imbalance across disciplines urgently needs to be addressed before the quality of medical education deteriorates irreversibly.

Many other African countries face faculty shortages. The 2009 Sub-Saharan Medical School Study found that 25% of responding medical schools had fewer than 50 total faculty (10). Responding medical schools reported an average of 29% of positions as vacant; schools perceived that a shortage of basic science teachers was a critical barrier to improving the quality of medical education (10).

The Lancet Commission on the future of health professionals’ education recommends major reforms in teaching and curricula (13), but this transformation requires time and development of capacity to deliver the new programs. To maintain and improve standards of medical education in Tanzania, it is imperative that there also be national agreement among medical schools and the ministries of education, health, and finance about the number of students medical schools have the capacity to train and corresponding strategies to prepare, recruit, and retain enough teachers for the future.

This review highlights the need for more research into the availability and teaching capacity of faculty. Prospective studies could form the basis for predicting the increase in capacity required to meet national needs for physicians and for developing strategies to build faculty capacity. Studies could inform, for example, how best to identify students on admission and during their training as to who might become future faculty and then prepare them through extra training and mentorship; develop a recruitment and retention package that attracts graduates to become career faculty; provide faculty development and mentoring that supports faculty in becoming effective teachers; and how best to make arrangements for efficient use of faculty time by reducing administrative tasks and supporting faculty research efforts.

## Paper context

Despite high disease burdens, very few doctors serve the people of Tanzania. Its universities are rapidly increasing their admissions of medical students. We reviewed publically available data about the faculty numbers in five medical schools in Tanzania in 2011/12 and found severe shortages across specialities. Four years later, the number of student admissions across the country has doubled, increasing the need for faculty to maintain the quality of medical training in Tanzania.
